# Transmission/Disequilibrium Tests Incorporating Unaffected Offspring

**DOI:** 10.1371/journal.pone.0114892

**Published:** 2014-12-23

**Authors:** Qinyu Wei, Yuanli Chen, Zheng Zeng, Chang Shu, Lu Long, Jianhua Lu, Yangxin Huang, Ping Yin

**Affiliations:** 1 Department of Epidemiology and Biostatistics, School of Public Health, Tongji Medical College, Huazhong University of Science and Technology, Wuhan, China; 2 Department of Epidemiology and Biostatistics, College of Public Health, University of South Florida, Tampa, Florida, United States of America; Johns Hopkins Bloomberg School of Public Health, United States of America

## Abstract

We propose a new method for family-based tests of association and linkage called transmission/disequilibrium tests incorporating unaffected offspring (TDTU). This new approach, constructed based on transmission/disequilibrium tests for quantitative traits (QTDT), provides a natural extension of the transmission/disequilibrium test (TDT) to utilize transmission information from heterozygous parents to their unaffected offspring as well as the affected offspring from ascertained nuclear families. TDTU can be used in various study designs and can accommodate all types of independent nuclear families with at least one affected offspring. When the study sample contains only case-parent trios, the TDTU is equivalent to TDT. Informative-transmission disequilibrium test (i-TDT) and generalized disequilibrium test(GDT) are another two methods that can use information of both unaffected offspring and affected offspring. In contract to i-TDT and GDT, the test statistic of TDTU is simpler and more explicit, and can be implemented more easily. Through computer simulations, we demonstrate that power of the TDTU is slightly higher compared to i-TDT and GDT. All the three methods are more powerful than method that uses affected offspring only, suggesting that unaffected siblings also provide information about linkage and association.

## Introduction

Spielman et al. (1993) popularized the transmission/disequilibrium test (TDT) for linkage and association between the marker loci and disease loci for use in studies of families with at least one affected offspring and two parents [1]. The TDT is a useful method to locate disease genes associated with complex diseases and has the desired property without giving spurious significant results even if there is population stratification which can lead to associations in the absence of linkage (Ewens and Spielman, 1995) [2]. Many extensions and generalizations of the TDT have been developed. Martin et al. (1997) provided a test statistic, TDT_sp_, that employs the information on transmissions to both members of an affected sib pair and that is valid as a test of both linkage and association [3]. Sun et al. (1999) extended TDT to 1-TDT for use in families with only one of parents available (incomplete nuclear families) [4]. Since the TDT is served as a test for unequal transmission of alleles from the parents to affected offspring, it cannot be performed if the genotypic data for the parents are not available. Horvath and Laird (1998) introduced a discordant-sibship test, the sibship disequilibrium test (SDT), that is used to test data from all of the affected and unaffected siblings [5].

TDT and 1-TDT have the common feature where the offsprings in the data to be analyzed are affected. For diseases with low prevalence, however, it was rather difficult to collect the data. Chao-yu Guo et al. (2007) introduced a new strategy called the informative-transmission disequilibrium test (i-TDT), which uses transmission information from hererozygous parents to all of the affected and unaffected offsprings in ascertained nuclear families and provides a valid chi-square test for both linkage and association [6]. The i-TDT was proved to be more powerful than the Family-based tests of association and linkage that use unaffected sibs, covariates, and interactions (FBAT-o-e) (Lake et al., 2000; Lunetta et al., 2000) which is extended from FBAT (Rabinowitz and Laird, 2000) [7], [8]. Generalized disequilibrium test (GDT) proposed by WeiMin Chen et al. (2009) is another generalization of TDT-like family-based association methods which assesses the genotype difference of all discordant relative pairs in a family and makes use of information beyond first-degree relative pairs [9]. Transmission/disequilibrium tests for quantitative traits (QTDT) proposed by Sun et al. (2000) was used in studies of quantitative trait [10]. The TDT (Spielman et al., 1993) for qualitative traits, the TDT (Rabinovitz, 1997) for quantitative traits and the 1-TDT (Sun et al., 1999) are special cases of QTDT.

This paper is organized as follows. We first introduce our modification on the statistic of QTDT to accommodate studies of qualitative traits and propose a new class of TDT-type tests, transmission/disequilibrium tests incorporating unaffected offspring (TDTU), for use in studies of families with affected and/or unaffected offsprings. The proposed test is valuable and allows researchers full use of the transmission information. Second, we explore the validity and the power of the tests in simulation studies. Finally, we conclude that the new tests are more powerful and allow researchers full use of the available data in detecting linkage between a marker locus and a disease locus.

## Methods

### General assumptions and notation

Suppose we have 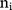
(i = 0, 1, 2) nuclear families with no, one, and two parents available in the study, respectively, where the subscript ‘i’ in 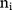 denotes the number of parents in a family. Let 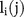 (i = 0, 1, 2; j = 1, 2, …, 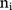) be the number of offsprings in the j-th family among families with i parents. We define the index function 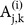 (k = 1,2,…,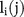)as 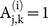 if the k-th offspring in the j-th family is affected, 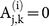 if the k-th offspring in the j-th family is not affected, and 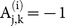 otherwise. We also assume that the marker locus of interest has two alleles ‘M’ and ‘m’. The parents and the offspring are genotyped at a marker locus of interest. For families with both parents, we define the following index function: 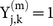 (or -1) if the mother in the j-th family is heterozygous and transmits the M (or m) allele to the k-th offspring, and 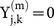 if the mother is homozygous. We similarly define 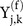 for the father. For families with one parent, we only consider offspring-parent pairs with genotypes (mM, mm) or (MM, mM), and offspring-parent pairs with genotypes (mm, mM) or (mM, MM). The first genotype in the bracket is the offspring's genotype and the second genotype is the available parent's genotype (Sun et al, 1999). We define the index function 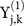 as 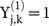 if the offspring-parent genotypes are (mM, mm) or (MM, mM), 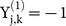 if the offspring-parent genotypes are (mm, mM) or (mM, MM), and 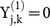 otherwise. Note that the index functions for families with two parents and for families with one parent differ by the superscript.

### TDTU when both parents are available

Rabinowitz (1997) first noted that, under the null hypothesis if no linkage between the marker locus and the quantitative loci, the trait value and the index functions 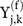 and 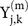 are conditionally independent, given the parental alleles. In the same way, we can conclude that conditional on the parental alleles, the index function 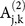 and the index functions 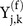 and 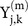 are conditionally independent, under the null hypothesis if no linkage between the marker locus and the disease locus. 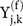 and 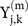 are independent under the null hypothesis, that is, 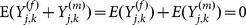


Thus for any constant c, 
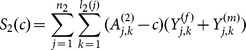
has mean 0.

The conditional variance of 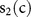 can be estimated by
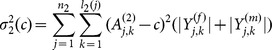



The class of statistics is given by
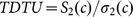
which has an approximate normal distribution when the number of heterozygous parents is large. When the number of families is small, simulations can be used to determine the p-value.

In this paper, let c be the incidence rate in the population. Let “D” and “d” denote the disease and normal allele. Let 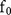, 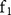, 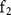 denote the probability of being affected when an individual carries 0, 1, 2 risk alleles (the phenocopy rate), and let Aff denote that an individual is diseased. Then c can be calculated by
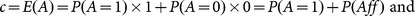


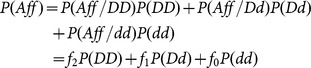


### 1-TDTU when only one parent is available

Sun et al. (1999) developed two test statistics for affected offspring applicable to families with one parent. In this paper, we extend those test statistics to affected and unaffected offspring. As in Sun et al. (1999), we assume that the probability of a missing parent is the same for all genotypes at the marker locus of interest. The first class of tests is applicable when either of the following two assumptions, A1 or A2 holds. The second class of tests is applicable even if both assumptions A1 and A2 are violated. However, the second class of tests is, in general, less powerful than the first class of tests.

Assumption A1: Males and females with the same genotype at the marker locus have the same mating preference,

Assumption A2: Both father and mother in each nuclear family are missing with the same probability 1/2 given one of them is missing.

#### 1-TDTU when assumption A1 or assumption A2 holds

Under the null hypothesis of no linkage between the marker locus and the disease locus, 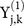 are independent of 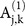. Sun et al. (1999) showed that 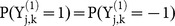 under the null hypothesis of no linkage if assumption A1 or A2 holds. Thus, for any constant c, the conditional mean of 
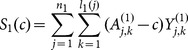
is zero. Unlike the situation when both parents are available, here (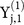, 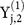,…,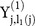) are not independent. The variance of 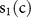 can be estimated by 
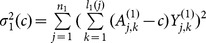
. The class of statistical tests for the null hypothesis is given by


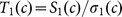
 Note that 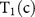 is the same as the first test statistic in Sun et al. (1999) for any c≠1.

#### The 1-TDT when both assumptions A1 and A2 are violated

When both assumptions A1 and A2 are violated, 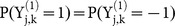 no longer holds in general even under the null hypothesis of no linkage between the marker locus and the disease locus. Following Sun et al. (1999), we modify 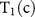 as follows. First, we calculate 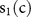 and 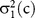 using families with father available. The corresponding values are denoted by 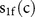 and 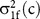, respectively. Similarly, we calculate 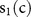 and 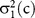 using families with mother available and the corresponding values are denoted by 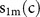 and 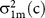, respectively. Let 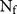 and 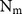 be the numbers of families with father and mother available, respectively. Define
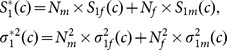



The new class of test statistics is then given by
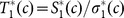



### Simulations

In this section, we evaluate the validity and the power of TDTU, i-TDT, GDT and TDT through simulation studies. All the simulations are accomplished with R2.14.1. We considered additive, recessive, and dominant disease models, and simulated two populations. In the first one, every nuclear family has exactly two offsprings; in the other, every nuclear family has exactly three offsprings. Each population consisted of two sub-populations, and a family belongs to one of them with equal probability 0.5. Under each population, we randomly selected 200 nuclear families with at least one affected offspring under various disease models and different sub-populations. Hence, in the first population, ascertained nuclear families have either 2 or 1 affected offspring; and in the second population, there would be 3, 2, or 1 affected offspring.

The transmissions from a parent to its affected offspring are correlated if there is linkage, even if there is no association (Spielman and Ewens, 1996). So as a test for linkage TDT is valid for any number of affected offspring in the nuclear families. However, as a test for association either the parental alleles are counted only once or the dependence among offsprings must be allowed in the analysis. Thus, for families with two affected offsprings, we randomly select one to incorporate into analyses for it. TDT ignores information from parents to unaffected offspring. The TDTU, i-TDT and GDT use all transmission information from parents to the offspring regardless of the affection status of the offspring.

We assume that the marker locus of interest has two alleles “M” and “m”. Let “D” and “d” denote the disease and normal allele. Then there are 4 possible haplotypes, which can be denoted as MD, Md, mD, md with corresponding relative frequencies p1, p2, p3, p4, respectively. Let 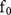, 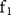, 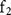 denote the probability of being affected when an individual carries 0, 1, 2 risk alleles (the phenocopy rate), respectively. We examined a range of possible values for the disequilibrium coefficient δ = p(DM)-p(D)p(M). The recombination fraction θ = 0.001. The disease model, phenocopy rate, and the penetrance are indicated in each figure. The additive and recessive results were similar(not shown here).

We simulated 10,000 replicates to check the type-I error at the significance level α = 0.05, and 2,000 replicates for the power comparisons. For simulations under the null hypothesis of no linkage or no association, the fraction of times that each test statistic exceeds the critical value is the type-I error. The power of each model is the proportion of significant test under the alternative hypothesis.

## Results

We consider three comparisons corresponding to the last three columns in Table 1 in S1 Appendix separately. The first comparison for the four statistics is no association in the presence of linkage; the second one is no linkage in the presence of association, and the last one is no association and no linkage. The TDTU, i-TDT, GDT and TDT all have correct type-I error for testing these three types of composite hypotheses. We can see that, all the three statistics have correct type- I error for testing these three types of composite hypotheses, suggesting that they are valid for both linkage and association.

**Figure 1:**
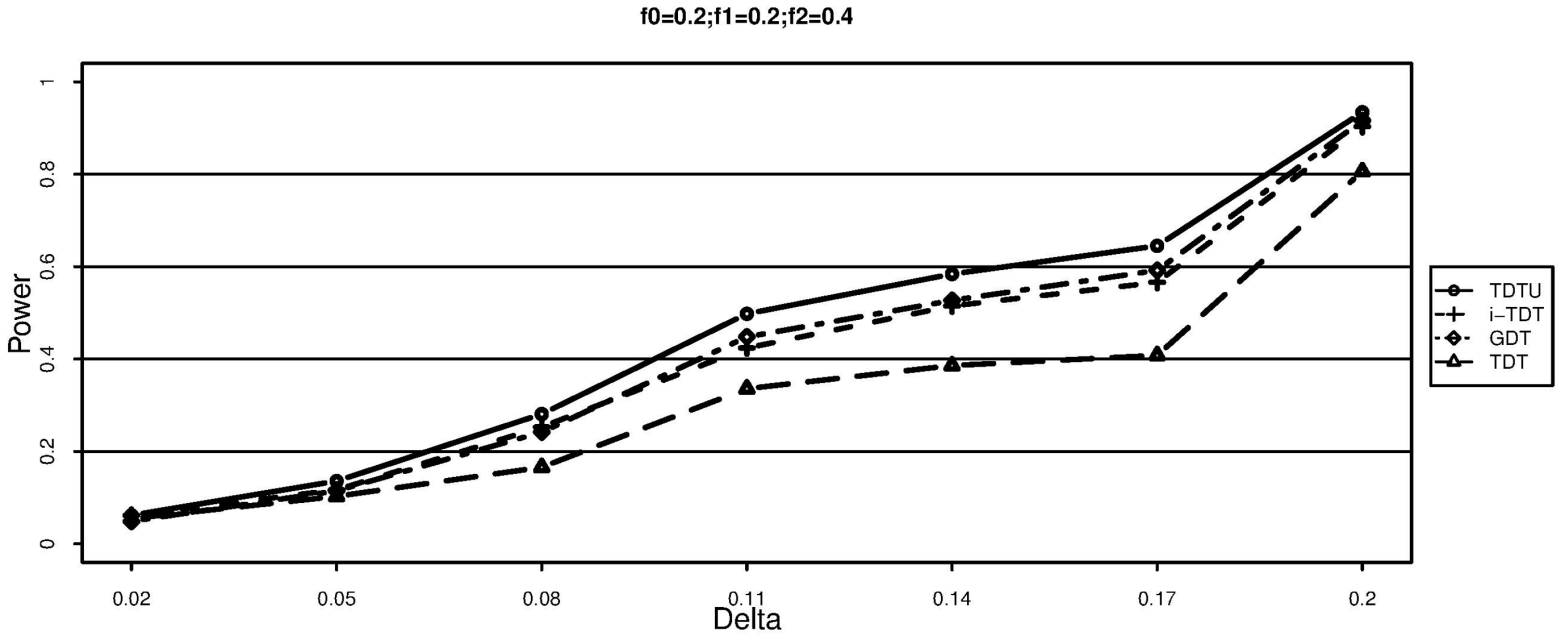
Power (each family has two offsprings).

**Figure 2:**
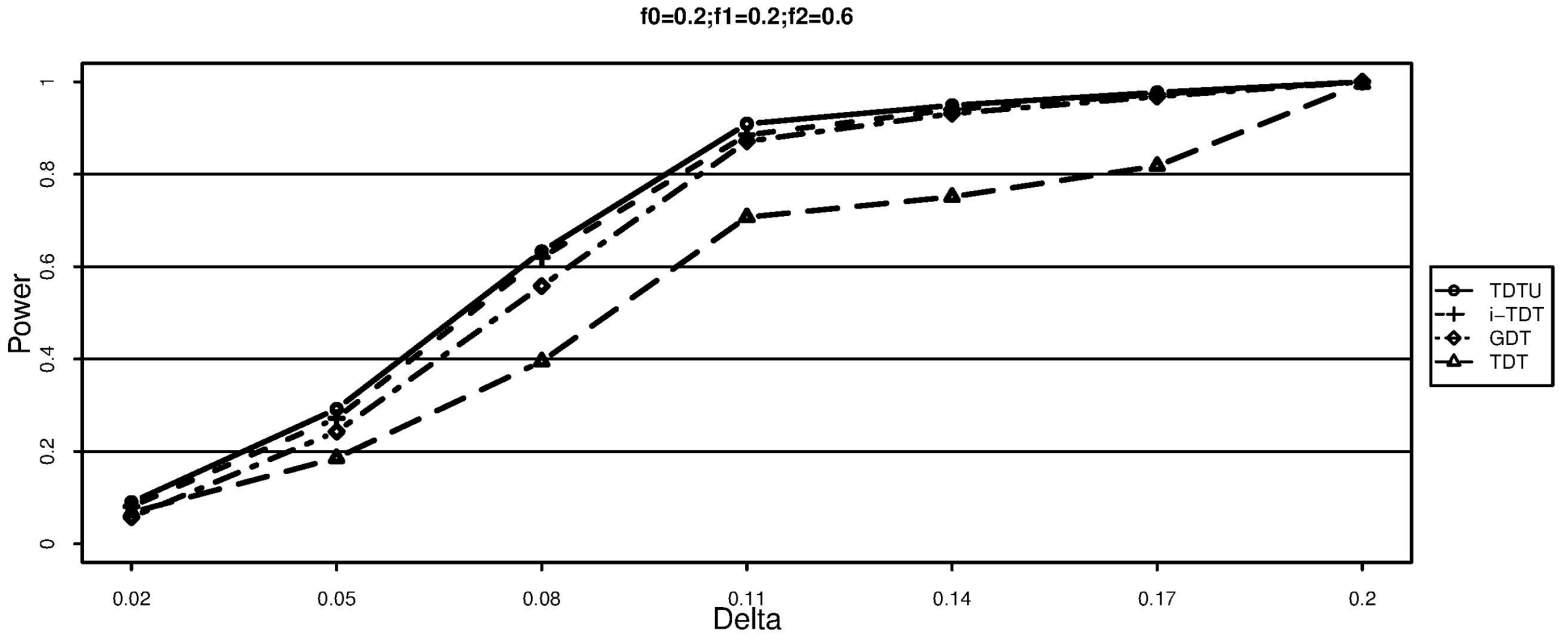
Power (each family has two offsprings).

The simulation results for additive disease models are shown in Figs. 1 and 2. It can be seen that TDT is significantly less powerful than TDTU, i-TDT and GDT due to loss of information. In addition, TDTU is consistently more powerful than i-TDT and GDT.

**Figure 3:**
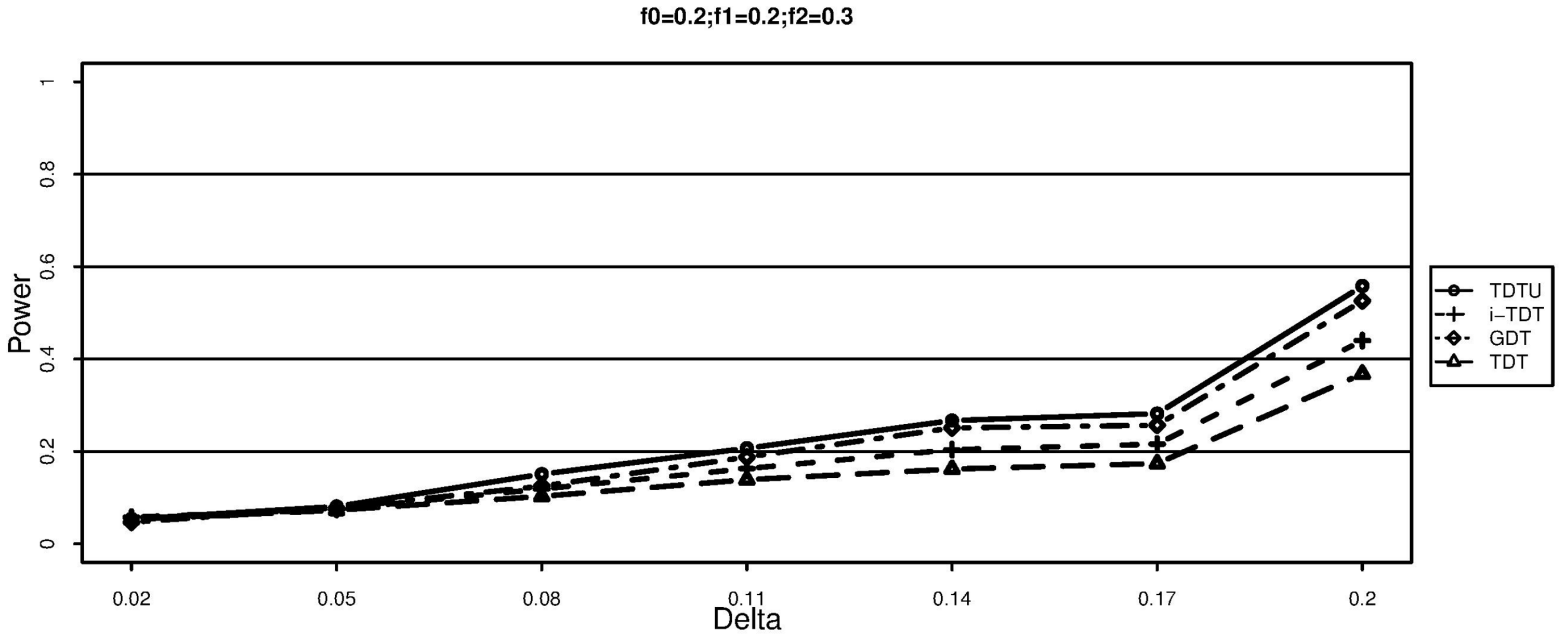
Power (each family has three offsprings).

**Figure 4:**
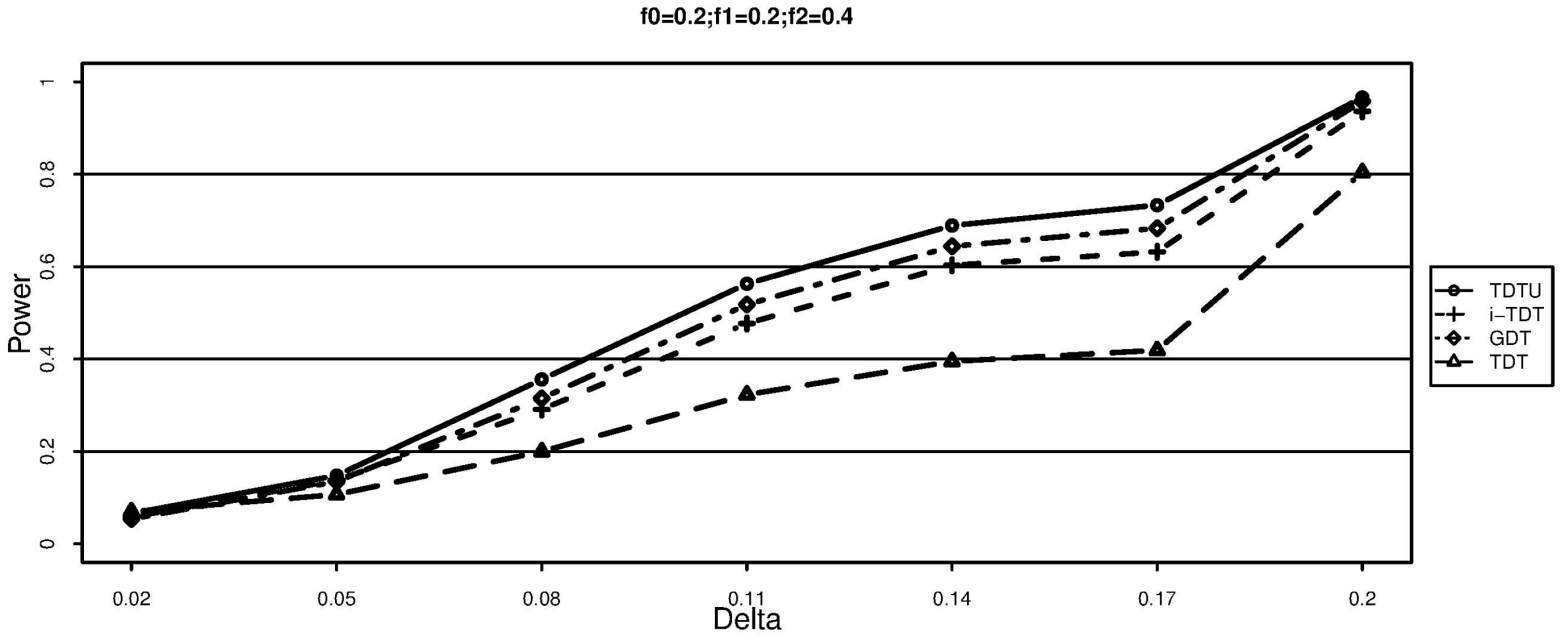
Power (each family has three offsprings).

**Figure 5:**
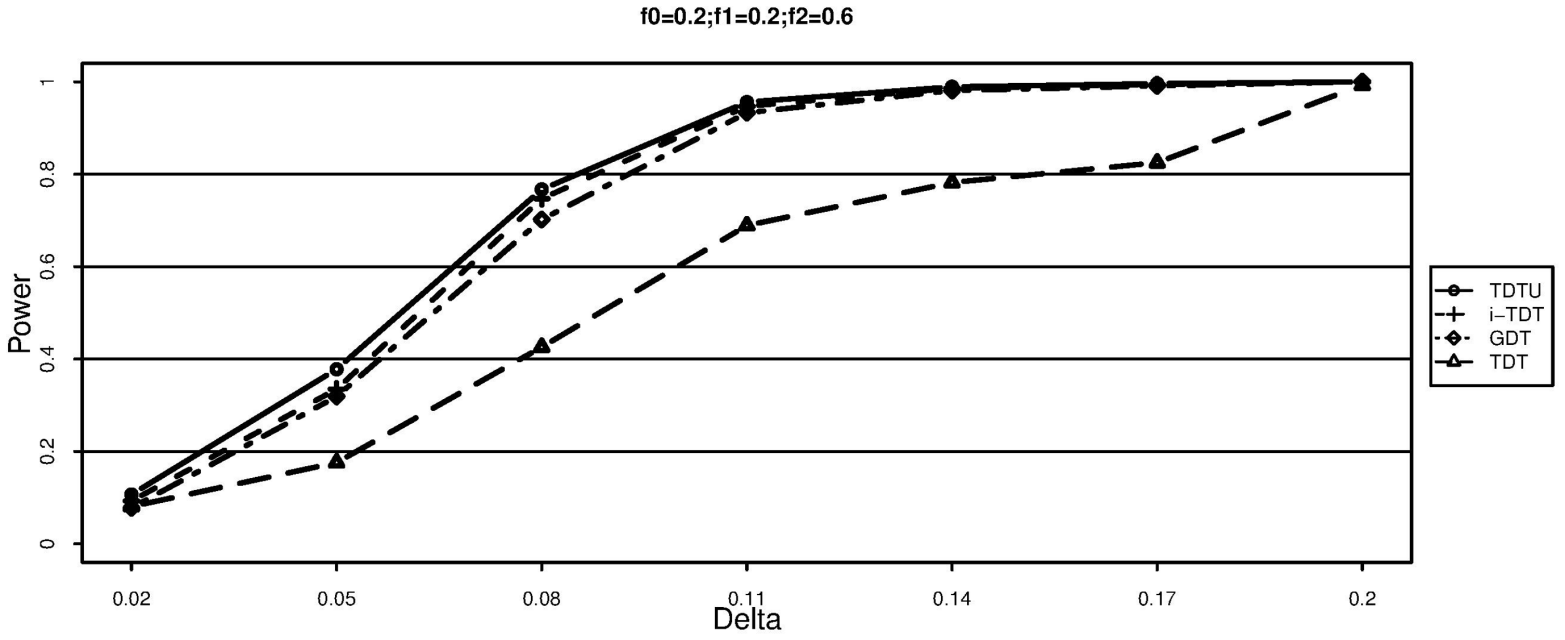
Power (each family has three offsprings).

From Figs. 3–5, we can draw the same conclusion with Figs. 1–2. We can also see that the powers of TDTU, i-TDT and GDT in Figs. 3–5 are higher than that in Figs. 1–2 because of the difference of sample size caused by the offspring number in each family. TDT doesn't get much change since we randomly selected one affected offspring from each family for it. The additive and recessive results are similar (not shown here); more details about the results from simulation studies can be found from tables 1–8 in the S1 Appendix.

## Concluding Discussion

In this paper, we first propose a new method for family-based tests of association and linkage called transmission/disequilibrium tests incorporating unaffected offspring (TDTU), which is constructed based on transmission/disequilibrium tests for quantitative traits (QTDT). TDTU can utilize transmission information from heterozygous parents to both affected and unaffected offspring regardless of sibship size and affection status. TDTU can be used in various study designs and can accommodate all types of independent nuclear families with at least one affected offspring. When the study sample contains only case-parent trios, the TDTU is equivalent to TDT. In contract to i-TDT, another method that uses information of both unaffected offspring and affected offspring, the test statistic of TDTU is simpler and more explicit, and can be implemented more easily. From the simulation results above, we can see that power of the TDTU is slightly higher compared to i-TDT and GDT. All TDTU, i-TDT and GDT are more powerful than TDT which uses affected offspring only, suggesting that unaffected siblings also provide information about linkage and association.

## Supporting Information

S1 Appendix (DOCX)
