# Supplementary material for: Transmission/Disequilibrium Tests Incorporating Unaffected Offspring
Source: PLoS One. 2014 Dec 23;9(12):e114892. doi: 10.1371/journal.pone.0114892 (PMC4275232; doi:10.1371/journal.pone.0114892)
Supplement: S1 Appendix — (DOCX) [file pone.0114892.s001.docx]

**Appendix**

In this appendix, we provide the detailed information of the power based on the four methods under various situations. Tables 2-4 are the results calculated from the data where each family contains two offsprings. Tables 5-8 are the results calculated from the data where each family has three offsprings.

**Tables**

TABLE 1. Type-I error

|  | δ=θ=0 | δ=0.05;θ=0.5 | δ=0;θ=0.5 |
| --- | --- | --- | --- |
| TDTU | 0.0539 | 0.0495 | 0.0452 |
| i-TDT | 0.0508 | 0.0510 | 0.0504 |
| GDT | 0.0431 | 0.0376 | 0.0399 |
| TDT | 0.0466 | 0.0499 | 0.0475 |

TABLE 2. Power based on the three methods with different parameters

|  | *f*_0_=*f*_1_=0.2, *f*_2_=0.4 | | | | *f*_0_=*f*_1_=0.2, *f*_2_=0.6 | | | |
| --- | --- | --- | --- | --- | --- | --- | --- | --- |
| *δ* | TDTU | i-TDT | GDT | TDT | TDTU | i-TDT | GDT | TDT |
| 0.02 | 0.062 | 0.062 | 0.049 | 0.055 | 0.090 | 0.081 | 0.058 | 0.068 |
| 0.05 | 0.136 | 0.117 | 0.115 | 0.103 | 0.292 | 0.272 | 0.243 | 0.186 |
| 0.08 | 0.281 | 0.253 | 0.242 | 0.166 | 0.633 | 0.620 | 0.558 | 0.395 |
| 0.11 | 0.498 | 0.424 | 0.448 | 0.336 | 0.909 | 0.885 | 0.871 | 0.707 |
| 0.14 | 0.584 | 0.515 | 0.527 | 0.386 | 0.949 | 0.940 | 0.931 | 0.751 |
| 0.17 | 0.645 | 0.566 | 0.592 | 0.408 | 0.977 | 0.973 | 0.968 | 0.818 |
| 0.2 | 0.934 | 0.903 | 0.916 | 0.806 | 1.000 | 1.000 | 1.000 | 0.996 |

Each family has two offsprings, and at least one affected.

TABLE 3. Power based on the three methods with different parameters

|  | *f*_0_=0.2, *f*_1_=*f*_2_=0.4 | | | | *f*_0_=0.2, *f*_1_=*f*_2_=0.6 | | | |
| --- | --- | --- | --- | --- | --- | --- | --- | --- |
| *δ* | TDTU | i-TDT | GDT | TDT | TDTU | i-TDT | GDT | TDT |
| 0.02 | 0.052 | 0.051 | 0.029 | 0.042 | 0.052 | 0.046 | 0.033 | 0.053 |
| 0.05 | 0.077 | 0.074 | 0.060 | 0.066 | 0.129 | 0.126 | 0.076 | 0.087 |
| 0.08 | 0.081 | 0.081 | 0.062 | 0.067 | 0.139 | 0.135 | 0.086 | 0.103 |
| 0.11 | 0.193 | 0.183 | 0.160 | 0.138 | 0.407 | 0.399 | 0.301 | 0.215 |
| 0.14 | 0.191 | 0.173 | 0.147 | 0.142 | 0.449 | 0.452 | 0.310 | 0.227 |
| 0.17 | 0.397 | 0.365 | 0.341 | 0.269 | 0.801 | 0.797 | 0.688 | 0.485 |
| 0.2 | 0.338 | 0.307 | 0.280 | 0.194 | 0.706 | 0.699 | 0.583 | 0.340 |

Each family has two offsprings, and at least one affected.

TABLE 4. Power of the three methods with different parameter

|  | *f*_0_=0.2, *f*_1_=0.3, *f*_2_=0.6 | | | | *f*_0_=0.2, *f*_1_=0.4, *f*_2_=0.8 | | | |
| --- | --- | --- | --- | --- | --- | --- | --- | --- |
| *δ* | TDTU | i-TDT | GDT | TDT | TDTU | i-TDT | GDT | TDT |
| 0.02 | 0.079 | 0.069 | 0.050 | 0.067 | 0.099 | 0.094 | 0.057 | 0.067 |
| 0.05 | 0.221 | 0.204 | 0.174 | 0.143 | 0.336 | 0.337 | 0.227 | 0.174 |
| 0.08 | 0.448 | 0.449 | 0.367 | 0.258 | 0.619 | 0.659 | 0.492 | 0.307 |
| 0.11 | 0.785 | 0.774 | 0.721 | 0.532 | 0.942 | 0.952 | 0.888 | 0.644 |
| 0.14 | 0.853 | 0.850 | 0.795 | 0.576 | 0.970 | 0.981 | 0.929 | 0.682 |
| 0.17 | 0.946 | 0.944 | 0.919 | 0.720 | 0.997 | 0.998 | 0.990 | 0.835 |
| 0.2 | 1.000 | 0.998 | 0.999 | 0.945 | 1.000 | 1.000 | 1.000 | 0.975 |

Each family has two children, and at least one affected.

TABLE 5. Power based on the three methods with different parameters

|  | *f*_0_=*f*_1_=0.2, *f*_2_=0.3 | | | | *f*_0_=*f*_1_=0.2, *f*_2_=0.4 | | | |
| --- | --- | --- | --- | --- | --- | --- | --- | --- |
| *δ* | TDTU | i-TDT | GDT | TDT | TDTU | i-TDT | GDT | TDT |
| 0.02 | 0.054 | 0.058 | 0.047 | 0.053 | 0.066 | 0.058 | 0.054 | 0.070 |
| 0.05 | 0.081 | 0.075 | 0.078 | 0.073 | 0.148 | 0.138 | 0.135 | 0.107 |
| 0.08 | 0.151 | 0.119 | 0.125 | 0.103 | 0.356 | 0.291 | 0.315 | 0.200 |
| 0.11 | 0.207 | 0.163 | 0.188 | 0.139 | 0.563 | 0.477 | 0.518 | 0.323 |
| 0.14 | 0.267 | 0.204 | 0.251 | 0.162 | 0.689 | 0.603 | 0.644 | 0.395 |
| 0.17 | 0.282 | 0.216 | 0.257 | 0.174 | 0.733 | 0.632 | 0.683 | 0.419 |
| 0.2 | 0.558 | 0.440 | 0.526 | 0.368 | 0.966 | 0.936 | 0.958 | 0.804 |

Each family has three children, and at least one affected.

TABLE 6. Power based on the three methods with different parameters

|  | *f*_0_=*f*_1_=0.2, *f*_2_=0.6 | | | | *f*_0_=*f*_1_=0.3, *f*_2_=0.6 | | | |
| --- | --- | --- | --- | --- | --- | --- | --- | --- |
| *δ* | TDTU | i-TDT | GDT | TDT | TDTU | i-TDT | GDT | TDT |
| 0.02 | 0.107 | 0.093 | 0.078 | 0.081 | 0.077 | 0.067 | 0.054 | 0.063 |
| 0.05 | 0.378 | 0.335 | 0.319 | 0.176 | 0.202 | 0.188 | 0.155 | 0.102 |
| 0.08 | 0.767 | 0.747 | 0.702 | 0.426 | 0.496 | 0.494 | 0.391 | 0.207 |
| 0.11 | 0.956 | 0.947 | 0.933 | 0.689 | 0.721 | 0.701 | 0.617 | 0.321 |
| 0.14 | 0.989 | 0.986 | 0.981 | 0.782 | 0.828 | 0.817 | 0.754 | 0.385 |
| 0.17 | 0.996 | 0.994 | 0.991 | 0.825 | 0.866 | 0.842 | 0.79 | 0.422 |
| 0.2 | 1.000 | 1.000 | 1.000 | 0.993 | 0.996 | 0.996 | 0.989 | 0.802 |

Each family has three children, and at least one affected.

TABLE 7. Power based on the three methods with different parameters

|  | *f*_0_=0.2, *f*_1_=*f*_2_=0.4 | | | | *f*_0_=0.2, *f*_1_=*f*_2_=0.6 | | | |
| --- | --- | --- | --- | --- | --- | --- | --- | --- |
| *δ* | TDTU | i-TDT | GDT | TDT | TDTU | i-TDT | GDT | TDT |
| 0.02 | 0.052 | 0.048 | 0.041 | 0.060 | 0.054 | 0.049 | 0.032 | 0.054 |
| 0.05 | 0.090 | 0.081 | 0.071 | 0.071 | 0.187 | 0.176 | 0.117 | 0.086 |
| 0.08 | 0.100 | 0.091 | 0.072 | 0.072 | 0.212 | 0.207 | 0.130 | 0.105 |
| 0.11 | 0.235 | 0.209 | 0.200 | 0.129 | 0.582 | 0.584 | 0.447 | 0.223 |
| 0.14 | 0.253 | 0.223 | 0.207 | 0.153 | 0.642 | 0.641 | 0.492 | 0.264 |
| 0.17 | 0.529 | 0.490 | 0.469 | 0.297 | 0.945 | 0.941 | 0.879 | 0.558 |
| 0.2 | 0.442 | 0.398 | 0.382 | 0.191 | 0.883 | 0.884 | 0.786 | 0.370 |
|  |  |  |  |  |  |  |  |  |

Each family has three offsprings, and at least one affected.

TABLE 8. Power based on the three methods with different parameters

|  | *f*_0_=0.2, *f*_1_=0.3, *f*_2_=0.6 | | | | *f*_0_=0.2, *f*_1_=0.4, *f*_2_=0.8 | | | |
| --- | --- | --- | --- | --- | --- | --- | --- | --- |
| *δ* | TDTU | i-TDT | GDT | TDT | TDTU | i-TDT | GDT | TDT |
| 0.02 | 0.092 | 0.075 | 0.065 | 0.070 | 0.135 | 0.121 | 0.068 | 0.072 |
| 0.05 | 0.300 | 0.277 | 0.241 | 0.139 | 0.472 | 0.476 | 0.363 | 0.180 |
| 0.08 | 0.560 | 0.607 | 0.527 | 0.281 | 0.819 | 0.856 | 0.714 | 0.337 |
| 0.11 | 0.887 | 0.870 | 0.840 | 0.504 | 0.989 | 0.990 | 0.973 | 0.640 |
| 0.14 | 0.943 | 0.941 | 0.918 | 0.589 | 0.997 | 0.999 | 0.993 | 0.724 |
| 0.17 | 0.983 | 0.979 | 0.971 | 0.734 | 1.000 | 1.000 | 1.000 | 0.871 |
| 0.2 | 1.000 | 1.000 | 1.000 | 0.944 | 1.000 | 1.000 | 1.000 | 0.975 |

Each family has three offsprings, and at least one affected.
